# German cancer statistics 2004

**DOI:** 10.1186/1471-2407-10-52

**Published:** 2010-02-22

**Authors:** Jörg Haberland, Joachim Bertz, Ute Wolf, Thomas Ziese, Bärbel-Maria Kurth

**Affiliations:** 1 Robert Koch Institute, Department of Epidemiology and Health Reporting, Papestraße 62-66, 12101 Berlin, Germany

## Abstract

**Background:**

For years the Robert Koch Institute (RKI) has been annually pooling and reviewing the data from the German population-based cancer registries and evaluating them together with the cause-of-death statistics provided by the statistical offices. Traditionally, the RKI periodically estimates the number of new cancer cases in Germany on the basis of the available data from the regional cancer registries in which registration is complete; this figure, in turn, forms the basis for further important indicators.

**Methods:**

This article gives a brief overview of current indicators - such as incidence, prevalence, mortality, survival rates - on the most common types of cancer, as well as important ratios on the risks of developing and dying of cancer in Germany.

**Results:**

According to the latest estimate, there were a total of 436,500 new cancer cases in Germany in 2004. The most common cancer in men is prostate cancer with over 58,000 new cases per annum, followed by colorectal and lung cancer. In women, breast cancer remains the most common cancer with an estimated 57,000 new cases every year, also followed by colorectal cancer. These and further findings on selected cancer sites can be found in the current brochure on "Cancer in Germany", which is regularly published by the RKI together with the Association of Population-based Cancer Registries in Germany (GEKID). In addition, the RKI made cancer-prevalence estimates and calculated current morbidity and mortality risks at the federal level for the first time. According to these figures, the 5-year partial prevalence - i.e. the total number of cancer patients diagnosed over the past five years who are currently still living - exceeds 600,000 in men; the figure is about the same among women. Here, too, the most common cancers are prostate cancer in men and breast cancer in women. The lifetime risk of developing cancer, which is more related to the individual, is estimated to be higher among men (48.5%) than among women (40.3%). In roughly rounded figures, therefore, about every second person in Germany develops cancer in the course of their lives. One in four men and one in five women die of cancer.

**Conclusions:**

In recent years, population-based cancer registration in Germany has come significantly closer to the aim of the complete, nationwide coverage of cancer. The continuous improvements in the data situation help describe cancer development in Germany.

## Background

Since the Federal Cancer Surveillance Unit was founded in 1983, first the Federal Health Office and then the Robert Koch Institute (RKI) have been regularly pooling and reviewing the data from the German population-based cancer registries and evaluating them together with the cause-of-death statistics provided by the statistical offices. The Federal Cancer Registry Act, which came into force in 1995, made it obligatory for all federal states (*Länder*) to set up population-based cancer registries.

This law, which has expired in the meantime, gave the development of cancer registration in the *Länder *a major boost, with the result that a population-based cancer registry now exists in every state. The federal act also briefly described the tasks of the Federal Cancer Surveillance Unit: i.e. to pool and evaluate the population-based data from Germany's cancer registries, and to determine and regularly publish development trends and regional differences. In addition, the law stipulated that data from the regional population-based cancer registries must be sent to the Robert Koch Institute once a year. After the Act expired, this passage was taken over in most of the *Länder *cancer registry laws [1]. The last few federal states only recently laid the legal foundation for state-wide, population-based cancer registration.

The Association of Population-based Cancer Registries in Germany (GEKID) was formed in 2004. Its members include not only the German population-based cancer registries, but also a tumour centre and interested scientists working in the field of cancer epidemiology. This association's primary task is to standardize as far as possible the content and methodology of cancer registration, despite the differences in legislation between the *Länder *[2,3].

Since the usefulness of population-based data on the incidence of cancer depends primarily on how completely all new cases of cancer are registered, the Federal Cancer Surveillance Unit at the RKI regularly reviews the completeness and reliability of the population-based cancer registries in Germany. This level of quality has been constantly improving in many *Länder *over the last few years, with the result that the International Agency for Research on Cancer (IARC) recently integrated seven German cancer registries and their data into the current edition of "Cancer in Five Continents" (Volume IX). Traditionally, the RKI periodically estimates the number of newly diagnosed cancer patients in Germany on the basis of the data from regional population-based cancer registries in which registration is complete; in turn, these figures form the basis for other important indicators such as prevalence rates and cancer-risk estimates.

This article gives a brief overview of current indicators - such as incidence, prevalence, mortality, survival rates - on the most common types of cancer, as well as important ratios on the risks of developing and dying of cancer (morbidity and mortality risks) in Germany. Detailed information on incidence, mortality and survival rates for more cancer sites is available in the latest brochure on "Cancer in Germany" (6th edition 2008), which is published jointly every two years by the RKI and GEKID [2].

## Methods

### Data sources

The basis for the RKI's periodic estimate of the number of new cancer cases occurring in Germany is provided by the morbidity data from the German population-based cancer registries in which registration is complete, as well as regional and federal mortality data.

The current estimate is based on the epidemiological case records of the population-based cancer registries in Germany up to 2004, which the RKI receives every year in anonymized form. International and national experience has shown that it takes more than two years before the population-based cancer registries' records on many cancer types are virtually complete; this is how long it takes before subsequent data collection and all comparisons with the existing registry data and death certificates have been essentially completed. The national mortality data from the statistical offices are currently available up to 2006. Because of the need for comparability with the registry data, this article also only processes mortality data up to 2004.

The annually updated figures on the average population provided by the statistical offices are used as the reference variable for the cancer morbidity and mortality data and thus cover all people who are resident in Germany, regardless of their nationality. As part of its 11th coordinated population projection, Germany's Federal Statistical Office offers several variations of cutoff-date-related population data broken down by age and sex for the years from 2006 to 2050 [4]. The individual variations reflect different assumptions on the future development of fertility, life expectancy and immigration. The present article is based on the 1 W-1 version, which makes moderate assumptions on each of the three factors mentioned above.

Recoding between the ICD-9, ICD-10 and ICD-O classifications was necessary in some cases. This was done using a conversion tool issued by DIMDI (German Institute for Medical Documentation and Information) and the so-called "IARCcrgTools" provided by the International Agency for Research on Cancer (IARC), based in Lyon, France [5,6]. This program was also used to apply the definitions recommended by the IARC on the independence of different primary tumours in a single person, to ensure that cancer incidence is correctly calculated.

### Statistical analysis

The completeness of the population-based data from the German cancer registries analysed every year by the RKI is checked using a procedure that has been agreed with the registries [7]. As stated in the brochure "Cancer in Germany" [2], in addition to the cancer registries that have been established for many years there are also numerous more recently formed registries. In the Saarland, cancer cases have been recorded with a high degree of continuity and stability for over 40 years, so that these data meet international standards of quality and completeness. To check the completeness of the other German cancer registries, the RKI first estimates incidence in the relevant catchment area on the basis of data from the Saarland's cancer registry (reference region) and compares this figure with the data actually collected by the respective registry. The ratios of incidence and mortality in the reference region are used for each site and gender to estimate cancer incidence levels, taking into account mortality in the region studied. The incidence rates are fitted using a Poisson regression model, with mortality as the offset and time (year) as the independent variable [8]. The IARC also uses Poisson regression models with mortality as the offset in the context of the GloboCan programme to estimate worldwide cancer incidence, albeit with age and sex as independent variables [9]. Where the level of completeness of the examined cancer registry is found to be adequate, the data acquired in this way are added to a data pool for a new reference region. In a second step, every registry is again checked against the data pool in order to determine level of completeness. As mentioned above, the latest results of the completeness estimate, which are broken down by sex, cancer site, age and region, are available in the current brochure on "Cancer in Germany" [2]. The newly created data pool also forms the basis for the nationwide estimate of the number of new cancer cases. As in the regional incidence estimate, the observed number of new cancer cases in the data pool is projected to the national level, taking national mortality into account [10].

Not only the past, but also the future development of new cancer cases is of major importance for public health policy. It provides clues on the likely level of pressure on the health system. In order to judge the possible effects of demographic developments on the incidence of cancer in Germany, this article simplifies the situation by assuming that the estimated current age-specific cancer incidence rates will not change in the coming years. This makes it possible to focus on quantifying the consequences of the changes in the future population structure that are expected according to the Federal Statistical Office's current population projections.

In addition to the two basic indicators (incidence and mortality) used to describe the development of the disease at the population level, prevalence is of particular interest for research into healthcare provision to assess the demand for care facilities and to plan for requirements accordingly. Prevalence is defined as the total number of people in the resident population who currently have cancer - or have had the disease in the past. This figure thus estimates the number of people in a population who are using certain care facilities at the same time. The problem with this indicator is deciding on the date when the patients are considered cured, because a cancer patient can suffer a relapse even after many years without symptoms. Furthermore, different cancer patients use the various care facilities to varying degrees in the course of the disease. Data on the total number of people still living who have had cancer at some time in the past, or still have the disease today, is certainly of less practical importance for care research. This article, therefore, does not present overall figures on all prevalent cases, but what are known as partial prevalence. These describe the number of patients still living who had cancer no more than 1, 2, 3, 4 or 5 years ago. The method used to calculate the indicators is based on the IARC's approach in the context of the Globocan programme [9,11]. In addition to the number of new cases in past years, the calculation method requires detailed information on the survival expectations of patients with the respective cancer. The IARC does not estimate the required observed survival rates themselves, but calculates them indirectly from available relative survival rates. The RKI used period analyses to calculate the observed survival rates in Germany directly from data from the Saarland Cancer Registry [12]. Furthermore, relative survival rates are presented for the current period window (2000-2004) according to Hakulinen's method [13], using national German life tables. Like the EUROCARE-4 study [14], the RKI also age-standardized the relative survival rates to enable comparisons to be made with the results of this international study. The calculation was made with a Stata program from Dickman et al. [15]. Moreover, as in the EUROCARE-4 study, the relative survival rates of patients with different cancers were age-standardized using different standards [16].

In addition to the indicators mentioned above, which are of particular interest for administrative planning, this article also presents indicators on cancer-morbidity and cancer-mortality risk, which are easier to relate to individuals. In this context, the age-conditional risk describes the probability of a person of a given age developing or dying of cancer within a specified number of years. The lifetime risk is a special case of age-conditional risk and relates to the probability of a newborn child developing or dying of cancer within the course of his or her life. The method of calculating the indicators follows the approach of Fay et al. [17] and uses the DevCan program [18] of the National Cancer Institute of the USA

## Results

### Estimated incidence and mortality

**Figure 1:**
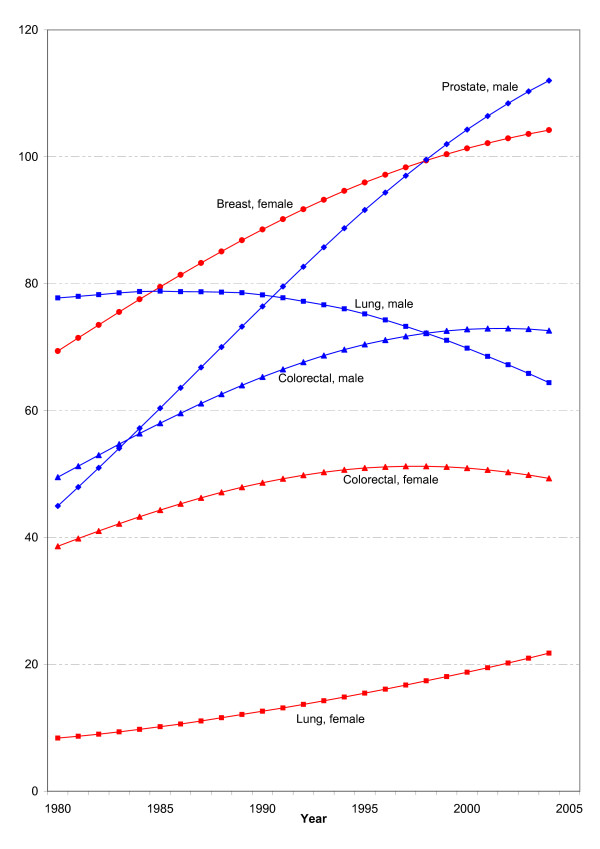
**Development of estimated age-standardized incidence rates (European population) of the most common cancer sites in men and women in Germany**.

Figure 1 shows long-term trends of the estimated age-standardized incidence rates for the three most common cancers in men and women for the period between 1980 and 2004 in Germany.

According to the RKI's current estimate, prostate cancer incidence showed the highest increase throughout the entire observation period. Prostate cancer, with currently about 58,000 new cases per annum, is now the most common cancer in men, followed by colorectal cancer (see Table 1). The incidence of lung cancer, which used to be the most common malignant neoplasm in men, has been declining markedly since the 1990s; the disease now occurs less frequently than colorectal cancer.

**Table 1:** Estimated number of new cancer cases and observed number of cancer deaths in Germany in 2004 (in brackets: age-standardized rates related to European population/2000 U.S. standard population).

Site *(ICD-10)*	Male		Female	
	*New Cases* *(Incidence rates)*	*Deaths* *(Mortality rates)*	*New Cases* *(Incidence rates)*	*Deaths* *(Mortality rates)*
**Colorectal ***(C18-C21)*	37,254 (72.6/78.0)	13,748 (26.9/31.4)	36,004 (49.3/55.1)	14,034 (17.0/20.7)

**Lung ***(C33, C34)*	32,848 (64.4/67.3)	28,820 (55.9/60.0)	13,188 (21.8/21.8)	11,026 (17.0/17.7)

**Breast ***(C50)*			57,231 (104.2/102.2)	17,592 (26.8/28.4)

**Prostate ***(C61)*	58,574 (112.0/117.7)	11,135 (22.2/29.6)		

**All cancer sites** *(C00-C97 except C44)*	230,464 (453.6/480.3)	110,745 (218.7/248.7)	206,010 (330.8/344.1)	98,079 (135.2/151.7)

In women, breast cancer remains the most common form of cancer with over 57,000 new cases in 2004, followed by colorectal cancer with about 36,000 cases. What is remarkable is the sharp increase in the rates of lung cancer among women, a trend that can also be observed in other European countries. According to the current estimate, the total percentage increase in the lung cancer incidence rate among women is 150% compared to 1980.

**Figure 2:**
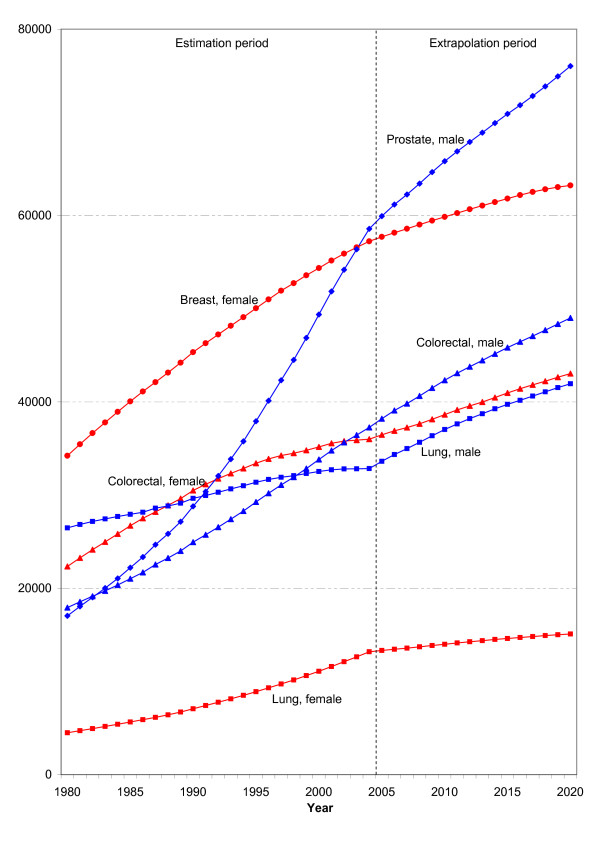
**Development of the estimated and extrapolated number of new cases of the most common cancer sites in men and women in Germany up to 2020**.

This means that, between 1980 to 2004, the age-standardized incidence rates for breast cancer rose to 150% and for prostate cancer to 250% of the respective figure for 1980 (see Figure 1). When the impact of demographic change is not offset by age standardization, the annual number of new cases of prostate cancer rises to as much as 340%, and for breast cancer to 170% of its initial value (see Figure 2).

**Figure 3:**
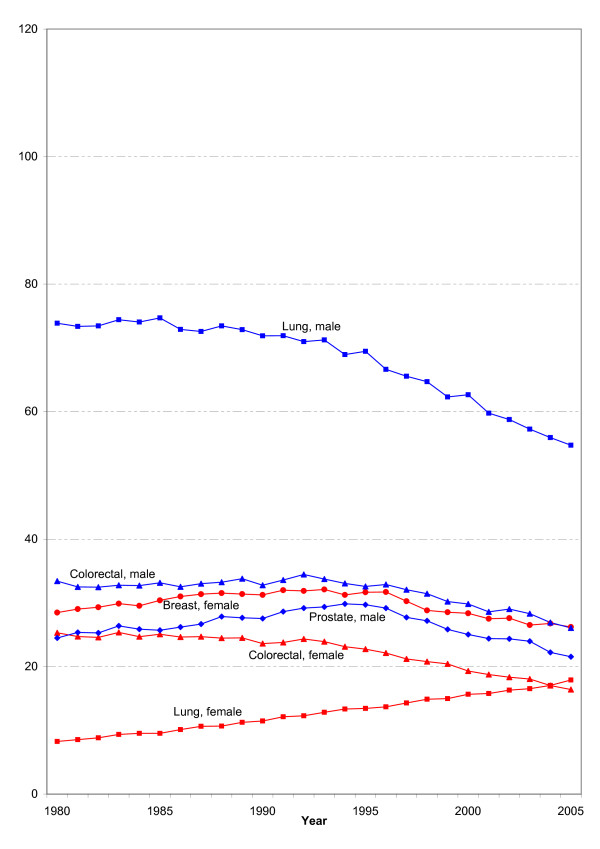
**Development of the age-standardized mortality rates (European population) of the most common cancer sites in men and women in Germany**.

Overall, the RKI currently estimates the number of new cancer cases in Germany at about 436,500 for 2004: 230,500 among men and 206,000 among women. This means an increase of more than 60% over 1980. Figure 3 shows the development of the age-standardized mortality rates of selected cancer sites. Only lung cancer mortality among women shows a sharp increase.

Figure 2 shows both the past development of new cancer cases and the extrapolated case figures up to 2020. In addition, Table 2 lists the corresponding age-specific new cases. On the simplifying assumption that the estimated current cancer incidence rates will remain constant in the future, the number of new cancer cases for all the sites observed here will rise sharply to a total of approximately 530,000 cases in 2020 - solely as a result of the anticipated demographic changes. Only in the under-45 age group will the future number of new cases for all cancer sites decline, since the Federal Statistical Office also expects a decline in this age group in its population projections up to 2020.

**Table 2:** Extrapolated number of new cancer cases in Germany up until 2020.

		Year		
Site *(ICD-10)*	Age group	*2010*	*2015*	*2020*
		*Male*		

**Colorectal ***(C18-C21)*	0-44	860	712	688
	45-64	11,228	12,631	13,496
	65-84	27,750	29,063	30,634
	85+	2,490	3,423	4,189
	All ages	42,328	45,829	49,007

**Lung ***(C33, C34)*	0-44	548	440	426
	45-64	11,425	12,782	13,552
	65-84	23,628	24,654	25,757
	85+	1,436	1,863	2,212
	All ages	37,037	39,739	41,947

**Prostate ***(C61)*	0-44	0	0	0
	45-64	16,604	18,928	20,805
	65-84	45,974	47,542	49,823
	85+	3,257	4,434	5,406
	All ages	65,835	70,904	76,034

**All cancer sites ***(C00-C97 except C44)*	0-44	11,019	9,736	9,406
	45-64	72,186	80,825	85,547
	65-84	163,502	171,026	179,207
	85+	12,108	16,321	19,780
	All ages	258,815	277,908	293,940

		*Female*		

**Colorectal ***(C18-C21)*	0-44	816	699	681
	45-64	7,627	8,488	8,799
	65-84	23,736	24,589	25,936
	85+	6,463	7,184	7,624
	All ages	38,642	40,960	43,040

**Lung ***(C33, C34)*	0-44	504	410	397
	45-64	5,096	5,610	5,762
	65-84	7,423	7,529	7,804
	85+	969	1,066	1,125
	All ages	13,992	14,615	15,088

**Breast ***(C50)*	0-44	5,678	4,763	4,632
	45-64	27,317	29,800	29,960
	65-84	23,696	23,744	24,929
	85+	3,170	3,512	3,718
	All ages	59,861	61,819	63,239

**All cancer sites ***(C00-C97 except C44)*	0-44	15,646	13,597	13,174
	45-64	66,973	73,654	75,050
	65-84	109,926	112,281	117,904
	85+	25,098	27,762	29,391
	All ages	217,643	227,294	235,519

### Survival rates

**Figure 4:**
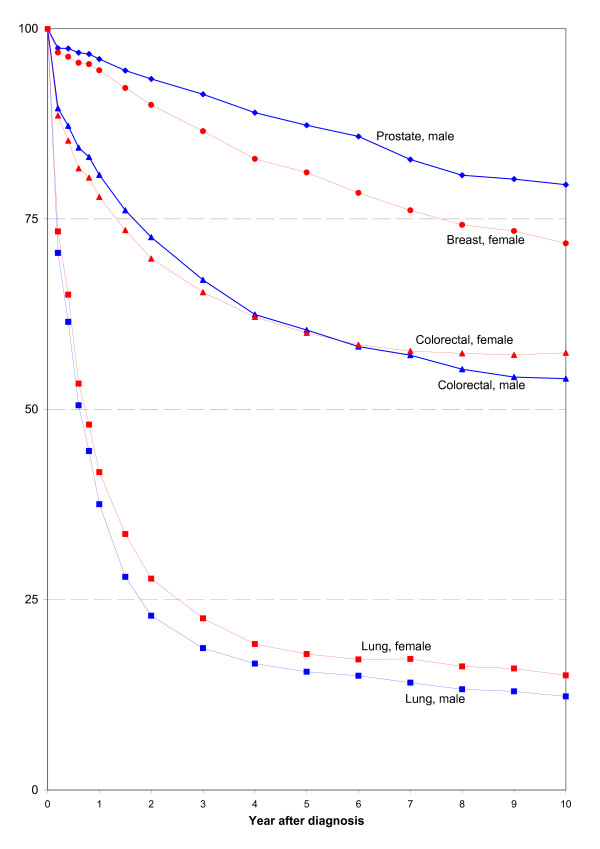
**Cumulated relative survival rates for selected cancer sites**.

Figure 4 shows the development of the cumulative relative survival rates of people suffering from the cancers covered in this article over the first 10 years after diagnosis. Of these sites, the survival expectations of male patients diagnosed with prostate cancer are the most favourable, followed by female patients with breast cancer. The survival expectations of patients with lung cancer are still very unfavourable for both sexes [19]. A comparison of the current 5-year relative survival rates calculated here using period analysis (period window 2000-2004) with the results of a traditional cohort analysis (diagnosis period 1993-1997) shows particularly striking improvements in the case of prostate cancer within this short time span [1].

Table 3 shows both the cumulative 5-year observed survival rates and the corresponding cumulative 5-year relative survival rates with and without age standardization. Age standardization facilitates international comparisons, for example with the results of the current EUROCARE-4 study, the results of which are based on the same age standards, or with the results of the CONCORD study of colorectal, breast and prostate cancer, which relate to the 1990-1994 diagnosis interval and include not only participating European countries, but also countries on all continents, for example the USA and Canada [14,20].

**Table 3:** Observed, relative and age-standardized relative 5-year survival rates (as %) including the 95% confidence intervals for the 2000-2004 period.

Site *(ICD-10)*	5-year survival rates		
	*observed*	*relative*	*age-stand. relative*
	*Male*		

**Colorectal ***(C18-C21)*	49.2 47.2 - 51.0	60.4 58.1 - 62.7	60.1 54.6 - 65.3

**Lung ***(C33, C34)*	13.0 11.8 - 14.2	15.5 14.1 - 17.0	15.4 12.1 - 19.2

**Prostate ***(C61)*	69.8 68.3 - 71.4	87.3 85.3 - 89.2	86.0 80.5 - 90.9

**All cancer sites** *(C00-C97 except C44)*	43.8 43.0 - 44.6	52.8 51.8 - 53.7	52.2 50.1 - 54.2

	*Female*		

**Colorectal ***(C18-C21)*	48.1 46.2 - 50.0	60.0 57.6 - 62.4	61.8 56.4 - 66.6

**Lung ***(C33, C34)*	15.8 13.6 - 18.2	17.8 15.3 - 20.5	16.6 11.8 - 22.5

**Breast ***(C50)*	72.5 71.2 - 73.8	81.1 79.6 - 82.5	79.6 76.1 - 82.8

**All cancer sites** *(C00-C97 except C44)*	51.3 50.5 - 52.1	59.9 59.0 - 60.9	58.3 56.4 - 60.2

### Prevalence

Table 4 shows the number of cancer cases by sex and site that occurred in Germany in 2004 and the previous years. A distinction was made between 1-, 3- and 5-year partial prevalence estimates. The corresponding percentages of the total population are given in brackets. According to these figures, in 2004 there were over 1.3 million people living in Germany who had had cancer during the 5 years between 2000 and 2004 (5-year partial prevalence), with a slightly higher proportion of women than men. Over one third of all prevalent cases in men were prostate cancer that had occurred no more than 5 years previously; in women, a third of the cases were breast cancer. The 5-year partial prevalence of colorectal cancer was virtually identical in men and women at about 110,000 cases respectively; incidence rates and survival rates were similar in both sexes. It may be surprising that the 1-year partial prevalence estimates were lower than the corresponding cancer incidence figures - not only in the case of colorectal cancer. However, while incidence indicates the total number of all cases of cancers that occurred between 1 January and 31 December each year, prevalence shows how many people had cancer on a certain date in the year - the cutoff date. Since not all patients survive the first year after diagnosis, the 1-year partial prevalence must always be lower than the respective incidence.

**Table 4:** Number of cancer cases in 2004 in Germany diagnosed over the last × years (percentages related to German population on cutoff date 31 Dec. 2004).

Site *(ICD-10)*	Number of cancer cases diagnosed over the last		
	*1 year*	*3 years*	*5 years*
	*Male*		

**Colorectal ***(C18-C21)*	32,400(0.08%)	79,350(0.20%)	114,509(0.28%)

**Lung ***(C33, C34)*	20,847(0.05%)	34,162(0.08%)	42,242(0.10%)

**Prostate ***(C61)*	56,025(0.14%)	149,497(0.37%)	222,254(0.55%)

**All cancer sites** *(C00-C97 except C44)*	202,261(0.50%)	456,087(1.13%)	644,302(1.60%)

	*Female*		

**Colorectal ***(C18-C21)*	31,131(0.07%)	76,988(0.18%)	113,208(0.27%)

**Lung ***(C33, C34)*	8,750(0.02%)	15,009(0.04%)	18,623(0.04%)

**Breast ***(C50)*	55,043(0.13%)	152,768(0.36%)	235,779(0.56%)

**All cancer sites** *(C00-C97 except C44)*	189,488(0.45%)	460,509(1.09%)	678,955(1.61%)

### Cancer risk

As Table 5 shows, approximately every second person in Germany develops cancer in the course of their life, although this roughly rounded result conceals the fact that the lifetime risk is higher for men than for women. On the other hand, only one in four men and one in every five women die of cancer (see Table 6).

**Table 5:** Probability of developing cancer within selected age intervals, by sex and selected sites, Germany.

	Age-conditional morbidity risk			
Site *(ICD-10*)	Birth to 49	50 to 69	70 and older	Lifetime morbidity risk
	*Male*			

**Colorectal ***(C18-C21)*	0.3%	3.3%	6.5%	8.0%

**Lung ***(C33, C34)*	0.3%	3.1%	5.1%	6.9%

**Prostate ***(C61)*	0.1%	5.5%	10.2%	12.4%

**All cancer sites** *(C00-C97 except C44)*	3.3%(1 in 31)	20.4%(1 in 5)	42.9% (1 in 2)	48.5%(1 in 2)

	*Female*			

**Colorectal ***(C18-C21)*	0.3%	2.1%	5.9%	7.3%

**Lung ***(C33, C34)*	0.2%	1.1%	1.4%	2.5%

**Breast ***(C50)*	2.0%	5.3%	4.5%	10.7%

**All cancer sites** *(C00-C97 except C44)*	4.8%(1 in 21)	15.5%(1 in 6)	28.5% (1 in 4)	40.3%(1 in 2)

**Table 6:** Probability of dying of cancer within selected age intervals, by sex and selected sites, Germany.

	Age-conditional mortality risk			
**Site ***(ICD-10)*	Birth to 49	50 to 69	70 and older	Lifetime mortality risk
	*Male*			

**Colorectal ***(C18-C21)*	0.1%	1.0%	3.1%	3.3%

**Lung ***(C33, C34)*	0.2%	2.5%	4.9%	6.2%

**Prostate ***(C61)*	0.0%	0.5%	3.8%	3.2%

**All cancer sites** *(C00-C97 except C44)*	1.0%(1 in 103)	8.3%(1 in 12)	22.9% (1 in 4)	25.7%(1 in 4)

	*Female*			

**Colorectal ***(C18-C21)*	0.1%	0.5%	2.8%	3.0%

**Lung ***(C33, C34)*	0.1%	0.8%	1.4%	2.2%

**Breast ***(C50)*	0.3%	1.2%	2.4%	3.5%

**All cancer sites** *(C00-C97 except C44)*	1.0%(1 in 97)	5.4%(1 in 18)	16.2% (1 in 6)	20.2%(1 in 5)

The highest cancer risk for men is prostate cancer, for women breast cancer. In the case of breast cancer, the risk is highest among middle-aged people, unlike the other sites observed where the risk of being diagnosed with the disease rises in old age. Compared to the last, corresponding RKI estimates for 2000, a 50-year-old woman's risk of developing breast cancer by her 70th year has now risen by one percentage point to 5.3% [1]. In men, the risk of being diagnosed with prostate cancer at this time of life has increased from 3.5% to currently 5.5%. Overall, the lifetime risk of developing the latter disease has risen since 2000 by about 3% to currently 12.4%.

## Discussion

The RKI's annual assessment of the completeness of the German cancer registry data shows that the coverage levels vary considerably from one cancer site to another. The data on a certain site from cancer registries deemed to be complete are merged and form the basis of the national estimates on cancer incidence. Coverage of breast cancer is the most complete of all the cancers studied here in all the registries. Concerning this cancer site the catchment areas of the participating cancer registries currently already cover 25% of the female population in Germany. Coverage of colorectal cancer, by contrast, is much worse: here, only 1.3% of the total population live in the catchment areas of the participating registries. The estimate on new cases of breast cancer is therefore based on a much more stable data foundation than other cancer sites.

Smoking has been recognized as a major risk factor for lung cancer for a long time. Levels of lung-cancer incidence and mortality largely reflect the population's past cigarette consumption. While tobacco consumption in Germany has decreased slightly among men since the mid-1980s, it is increasing among women. As a result, the smoking behaviour of the two sexes is increasingly converging [21], as reflected in decreasing lung-cancer incidence and mortality rates among men and rising rates among women.

The population's increased participation in screening tests is certainly one reason for the sharp increase in new cancer cases. A major factor in the case of men is blood testing for prostate-specific antigen (PSA), combined with earlier diagnosis [2,22,23]. The currently ongoing nationwide introduction of mammography screening for women has attracted considerable public attention and prompted several pilot projects in different regions of Germany in recent years. These have contributed to the sharp estimated increase in the number of new cases of breast cancer, although the most recent estimates indicate that the increase is no longer as steep as it has been in the past. Demographic changes (changes in the age structure) are another important cause of the increase. The extrapolations on the future development of cancer in Germany up to 2020 show that, even if cancer rates remain unchanged, the number of new cancer cases will grow significantly over the next few years, especially among men, solely as a result of the growing proportion of elderly people in the population that is anticipated in the future. However, a further study by the RKI also shows that the number of deaths from cancer could even fall in the following years, despite the above-mentioned demographic changes, if the trend towards declining mortality rates for all cancer sites that has been observed since the 1990s continues [24].

The survival expectations of men and women with the same cancer site differ only slightly overall, as shown by the overlapping 95% confidence intervals in the cumulative 5-year relative survival rates in Table 3. Overall, the significantly better survival rates for women with cancer are essentially based on gender-specific differences in the range of cancer sites - lung cancer being more common in men and breast cancer in women. The age-standardized survival rates shown in Table 3 are all above the respective average European figures given in the EUROCARE-4 study [14]. By contrast, with the exception of lung cancer, the North American SEER programme indicates much higher survival rates than the current rates calculated here [25]. If the same age standard as in the EUROCARE-4 study is used, as in the case of the CONCORD study, as early as the diagnosis years 1990-1994 this leads to only slightly less favourable results for colorectal cancer in the USA and Canada compared to the survival rates calculated by the RKI. In the case of breast cancer in women, the survival rates in the early 1990s in the USA and Canada are already slightly higher the current survival rate in Germany. The prostate-cancer survival rates in North America were already significantly better among patients diagnosed in the early 1990s; within Europe they were only comparable with the results from the Austrian state of Tyrol. The survival rates in Germany for the diagnosis years 2000-2004 presented in this paper correspond to those previously calculated for Canada and Austria [20].

Life expectancy in the Saarland is currently below the national average in both men and women [21]. Since the observed survival rates are currently still based exclusively on the survival experience of Saarland cancer patients, and since the rates are calculated taking into account all deaths, including deaths not caused by cancer, the reported survival rates probably slightly underestimate survival prospects of German cancer patients nationwide, with corresponding effects on the prevalence estimates.

The high morbidity risks, especially the lifetime risks - particularly in the case of prostate cancer in men and breast cancer in women as shown in Table 5 - must be seen in the context of the above-mentioned screening measures and therefore interpreted cautiously. It seems that it is not the risk of developing cancer that has increased, but the likelihood that such a disease will be detected and diagnosed at a younger age and an earlier stage of the disease. In both sites the increase in the morbidity risks is accompanied by a slight decline in mortality risks.

In general, when interpreting the above-mentioned morbidity and mortality risks, it should be borne in mind that the estimates made are based on the experience of the general population, and the only individual characteristics that are taken into account are gender and age. The morbidity and mortality risk of each individual can be higher or lower, depending on their personal behaviour, genetic predisposition and exposure to exogenous factors.

## Conclusions

In recent years, population-based cancer registration in Germany has come significantly closer to the aim of the complete, nationwide coverage of cancer. On the one hand, the data currently available in Germany help describe cancer development in the past; on the other, they provide the data for other predictions on possible developments in the field of cancer in Germany. In this context population-based survival rates represent an important indicator for evaluating the effectiveness of healthcare services. Estimates on the number of new cancer cases to be expected in the future are also an important aspect for requirements planning in healthcare. The continuous improvements in the data situation over the past few years make it possible, using simplifying assumptions, to indicate possible developments of cancer incidence in Germany up until 2020, both for cancer as a whole and for individual cancer sites. These can then be used to assess the effectiveness of certain public-health measures (e.g. screening programmes) in the fight against cancer and, where appropriate, to uncover areas where things are going wrong - in the interest of the individuals affected by a cancer diagnosis.

## Competing interests

The authors declare that they have no competing interests.

## Authors' contributions

JH drafted the manuscript with the support by the other authors. All authors read and approved the final manuscript.

## Pre-publication history

The pre-publication history for this paper can be accessed here:

http://www.biomedcentral.com/1471-2407/10/52/prepub
